# Antioxidants and Quality of Aging: Further Evidences for a Major Role of *TXNRD1* Gene Variability on Physical Performance at Old Age

**DOI:** 10.1155/2015/926067

**Published:** 2015-04-29

**Authors:** Serena Dato, Francesco De Rango, Paolina Crocco, Giuseppe Passarino, Giuseppina Rose

**Affiliations:** Department of Biology, Ecology and Earth Science, University of Calabria, 87036 Rende, Italy

## Abstract

Oxidative stress is a major determinant of human aging and common hallmark of age-related diseases. A protective role against free radicals accumulation was shown for thioredoxin reductase TrxR1, a key antioxidant selenoprotein. The variability of encoding gene (*TXNRD1*) was previously found associated with physical status at old age and extreme survival in a Danish cohort. To further investigate the influence of the gene variability on age-related physiological decline, we analyzed 9 tagging SNPs in relation to markers of physical (Activity of Daily Living, Hand Grip, Chair stand, and Walking) and cognitive (Mini Mental State Examination) status, in a Southern-Italian cohort of 64–107 aged individuals. We replicated the association of *TXNRD1* variability with physical performance, with three variants (rs4445711, rs1128446, and rs11111979) associated with physical functioning after 85 years of age (*p* < 0.022). In addition, we found two SNPs borderline influencing longevity (rs4964728 and rs7310505) in our cohort, the last associated with health status and survival in Northern Europeans too. Overall, the evidences of association in a different population here reported extend the proposed role of *TXNRD1* gene in modulating physical decline at extreme ages, further supporting the investigation of thioredoxin pathway in relation to the quality of human aging.

## 1. Introduction

The role of oxidative stress response in the susceptibility to longevity is a hot topic in aging research. Comparisons among species with different rates of aging suggested that long lived species tend to show reduced oxidative damage, reduced mitochondrial free radicals production, increased antioxidant defenses, and increased resistance to oxidative stress both* in vivo* and* in vitro* [1]. In humans, antioxidant defenses (i.e., plasma vitamins C and E) were suggested as key factors for healthy aging and longevity. Indeed, centenarians generally show a lower degree of oxidative stress [2]. Nevertheless, according to the oxidative stress hypothesis, oxygen-derived free radicals (ROS) are a primary causal factor underlying aging-associated declines in physiological functioning, increasing the incidence of diseases [3]. However, a direct cause-and-effect relationship between the accumulation of oxidative mediated damage and aging has not been strongly established. The overall cellular oxidative stress during aging is determined not only by ROS generation but also by a reduced defense capacity of antioxidant systems.

The thioredoxin system (thioredoxin (Trx), thioredoxin reductase (TrxR), and NADPH), a ubiquitous thiol oxidoreductase pathway, is a most important antioxidant frontier of the cell, able to regulate its reduction/oxidation (redox) status. Trx plays an essential role in the antioxidant defense, both directly, acting as redox regulator of intra- and extracellular signalling pathways and transcription factors, and indirectly, by protein-protein interactions with key signaling molecules such as thioredoxin-interacting protein (TXNIP). Furthermore, Trx protects the cell against lipid and protein peroxidation by controlling the protein folding through the catalysis of sulfur-exchange reactions among protein complexes [4, 5]. Its endogenous regulator, TrxR1, is a key selenoprotein antioxidant enzyme as well, able to reduce Trx (its main substrate) and other compounds, thus detoxifying cells from oxidative injuries [6, 7]. Highly conserved along the evolution, the system has also a pivotal role in growth promotion, neuroprotection, inflammatory modulation, antiapoptosis, immune function, and atherosclerosis [8, 9].

Mammalian Trxs were found implicated in several human diseases, including cardiovascular diseases, heart failure, stroke, inflammation, metabolic syndrome, arthritis, and cancer [9]. Their role has also been associated with neuroprotection against Alzheimer's and Parkinson's disease [10], so that their upregulation has been suggested as a good strategy for prevention and treatment of these disabling age-related diseases.

As for longevity, data on experimental organisms supports a role of this important antioxidant system in survival [11, 12]. In humans, thioredoxin system has been considered among* vitagenes* (a group of genes encoding for proteins involved in preserving cellular homeostasis during stressful conditions), together with heat shock proteins and the sirtuin protein systems [13]; however, its role in human aging remains unclear and poorly investigated.

An effect of this antioxidant system on human aging can be hypothesized from the association reported between genetic variability of the gene coding for TrxR1,* TXNRD1*, and late-life survival in a Northern European nonagenarian cohort [14]. Furthermore, a correlation with functional activity was demonstrated for some* TXNRD1* variants in the same Danish cohort [15], in particular with ADL (Activity of Daily Living), a geriatric parameter used to measure the ability to perform daily activities independently, which differentiates successfully aged individuals from those showing disabilities.

In the present work, we aimed to test the association of* TXNRD1* variability with health status and longevity found in Northern Europeans, by analyzing 9 common tagging SNPs in relation to markers of physical (ADL; Hand Grip (HG); Chair stand; and Walking) and cognitive (Mini Mental State Examination (MMSE)) status, in a Southern-Italian cohort, ranging from 64 to 107 years of age. Moreover, for verifying a possible effect of the gene variability on mortality, we tested the association with survival by a case-control approach.

## 2. Materials and Methods

### 2.1. Sample Study

The sample analyzed included 588 Southern-Italian subjects (323 females and 265 males), age range 64–107 years. All subjects were born in Calabria and their parents and grandparents were native of the same area; samples were collected within the framework of several recruitment campaigns carried out for monitoring the quality of aging in the whole Calabria region from 2002 onwards [16]. A multidimensional geriatric assessment was available for all the subjects, through a structured questionnaire administered during a home based interview by a trained operator. Questionnaire comprises a collection of sociodemographic information, sensory deficits, physical, cognitive, and depressive status, medications, and self-reported health status. Furthermore, physical tests such as Hand Grip (HG) strength, Chair stand, and ability to walk and physical measures such as height and weight were evaluated by a geriatrician. Subjects with dementia and/or neurologic disorders were not included. White blood cells (WBC) from blood buffy coats were used as source of DNA.

#### 2.1.1. Ethic Statement

Recruitment campaigns and subsequent analyses received the approval of the relevant ethical committees of the University of Calabria. All subjects provided written informed consent for the permission to collect blood samples and usage of register-based information for research purposes.

### 2.2. Geriatric Assessment

#### 2.2.1. Functional Activity

The management of activities of Daily Living (bathing, dressing, toileting, transfer from bed to chair, and feeding) was assessed using a modification of the Katz Index of ADL [17]. The assessment was based on what the subject was able to do at the time of the visit. The score is given counting the number of activities in which the participant is dependent or independent at the time of the visit. For the analyses, ADL scores were dichotomized as one if the subject was not independent in all five items and zero otherwise.

#### 2.2.2. Physical Performance

Hand Grip (HG) strength was measured by a handheld dynamometer (SMEDLEY's dynamometer TTM) while the subject was sitting with the arm close to his/her body. The test was repeated three times with the stronger hand and the maximum of these values was considered. Since HG strength is affected by age, sex, and height, the scores were corrected for these factors when necessary.

Chair stand test was carried out by asking the subject to stand up from and sit on a chair for 5 times, with arms folded, and registering the number of seconds spent to complete the test, measuring time lapsed by a chronometer.

Ability to walk was evaluated by a self-reported parameter, obtained by asking about the personal ability to walk for 500 meters. Usage of a cane or other supports was registered.

#### 2.2.3. Cognitive Performance

Mini Mental State Examination (MMSE) test was used to evaluate the cognitive performance in the analyzed sample. This 30-item questionnaire assesses orientation, episodic memory, attention, language, and construction functions [18]. Since it is affected by age and educational status, the MMSE scores were normalized for these variables according to a standardized procedure [19]. MMSE scores were dichotomized as zero if the subject showed a normal cognitive function (MMSE > 23) and one otherwise.

### 2.3. Genotyping

14 SNPs covering the whole* TXNRD1* genetic variability were prioritized by a tagging approach, attempting to choose those most likely to be of functional relevance (nonsynonymous SNPs, SNPs located in the 5′ and 3′ UTR regions). SNPs associated with health status and longevity in Northern Europeans [14, 15] were also chosen. SNPs with a minor allele frequency (MAF) less than 5% were excluded from the analysis.

Multiplex SNP genotyping was performed using iPLEX Gold Genotyping Assay and Sequenom MassARRAY (Sequenom, San Diego, CA, USA) according to manufacturer's instructions. Sequenom's MassARRAY Designer was used to design PCR and extension primers for each of the 14 SNPs selected. However, four of them (rs10861169, rs10861197, rs10047589, and rs4964287) were skipped by the software for primers design and were not analyzed in this paper. The details for genotyped SNPs are listed in Table 1.

### 2.4. Statistical Analyses

Allele frequencies were estimated by counting genes from the observed genotypes for each analyzed SNP and Hardy-Weinberg equilibrium (HWE) was verified by exact test [20].

The association between analyzed phenotypes and the variability of* TXNRD1* gene was tested by RobustSNP algorithm as proposed by So and Sham [21]. This is a robust association test based on a score test, very suitable for both quantitative and binary traits, which takes into account covariates. In the present study, the RobustSNP algorithm was applied to estimate the influence of genetic variability on the predisposition to both human longevity and a good functional status at old age. The whole sample was divided into long lived (349 subjects, 139 males and 210 females, age > 85 years) and younger controls (239 subjects, 126 males and 113 females, age ≤ 85 years), by using the 85 years of age as a cut-off for the classification according to biodemographic studies of mortality at older ages [22]. The different physical and cognitive parameters were analyzed separately in the two subgroups, due to the age specificity of most of geriatric measurements ([23] and references therein).

Different genetic models were tested (dominant, recessive, and additive). For each model, a *z*-score was returned, representing the *z*-statistics for the regression analyses, with a *p* ^Model^ referring to the *p* value for the most likely genetic model (dominant, recessive, and additive). Adjustment for multiple comparisons was performed: in particular, a theoretical combined *p* (theop) was obtained, representing the *p* value adjusted for multiple testing under the three genetic models tested. In the regression models used for testing the association between variability of analysed polymorphisms and human longevity, the variable sex was used as covariate. Age and sex were used when analyzing the association with functional parameters. The test has been implemented in the R package RobustSNP.

## 3. Results

Table 2 summarizes the main characteristics of the analyzed sample stratified by age group as previously defined.

One SNP, rs7962423, candidate because of an optimal tagger in the* TXNRD1* chromosomal region, did not pass the MAF limit of 5% in our population. All the remaining 9 SNPs followed the HWE in the control group (*p* > 0.05). A low call rate (27%) was found for the SNP rs4445711 in the control sample but not in long lived subjects (84% genotyping). Thus, case-control approach for longevity analyses was not applied to this variant, while association analyses with functional parameters in the oldest group can be considered for this SNP, because of being obtained without comparison between the two age classes.

No significant LD (*r* ^2^ < 0.8) was found between pairs of SNPs in the gene region (Figure S1) (see Figure S1 in Supplementary Material available online at http://dx.doi.org/10.1155/2015/926067), so single SNP instead of haplotype analysis was carried out.

### 3.1. *TXNRD1* Association with Functional and Cognitive Parameters

Complete results of the RobustSNP association tests are reported as Supplementary Material (Table S1). For each of the 9 analyzed SNPs, the three genetic models were tested by using the minor allele as reference and sex and age as covariates.  Table 3 reports the polymorphisms associated with functional performance at nominal significance (*p* ^Model^ < 0.05) level.

The most significant associations were found in the group of subjects older than 85 years of age. In particular, in long lived subjects, association was found between rs4445711 and ADL scores in a recessive way (*p* ^Model^ = 0.022), with subjects homozygote for the less frequent allele G showing higher disability level; likewise homozygosity for the same rs4445711-G allele was associated with reduced ability of Walking (*p* ^Model^ = 0.008). This parameter was found also associated with the rs11111979 in a recessive manner (*p* ^Model^ = 0.019). In the same age group, significant association with Chair stand score was found for the rs1128446 (*p* ^Model^ = 0.021 recessive), while only a trend of association was found with HG for the rs10861203 (*p* ^Model^ = 0.047 dominant). All these associations, except that with HG, were found significant also when correcting for multiple testing (theop < 0.05).

In younger controls, significant associations were detected with Chair stand and Walking parameters. The first is correlated with rs1128446 variability (*p* ^Model^ = 0.030) under a recessive model, consistently with that reported in long lived subjects, thus suggesting an effect of this genetic variability after 64 years of age and onwards; in particular, in both samples homozygote subjects for the less frequent allele G perform less in this physical test. Association with Walking in the control group concerns rs7310505 (*p* ^Model^ = 0.037 additive). All the associations in controls did not hold adjustment for multiple testing (theop > 0.05).

No correlation was found between* TXNRD1* variability and cognitive performances, in both age groups.

### 3.2. *TXNRD1* Association with Longevity

As reported in Table 4, only a trend of association with longevity was found for the SNPs rs4964728 and rs7310505 in the additive model (*p* ^Model^ = 0.041 and *p* ^Model^ = 0.072, resp.); for both SNPs the presence of the minor allele seems to negatively influence survival after 85 years of age.

## 4. Discussion

In experimental models, modification of stress response seems to characterize normal aging: a shift in the redox status with age, with a progressive change to a more prooxidant environment, was documented* in vivo* in many species, leading to an increase in ROS levels and a consequent aberrant regulation of redox-sensitive signaling pathways [24].

In humans, a relationship between increased oxidative stress and the most important age-related pathologies (i.e., cardiovascular and neurodegenerative diseases, cancer, and diabetes) was largely demonstrated ([25] and references therein), as well as association with frailty, a vulnerability status which exposes elderly individuals to a higher risk of poor outcomes, including infections, disabilities, institutionalization, and death [26].

Considering that the paradigm “less ROS formation = more years to live” is not fully true, because of the important role of ROS in cellular signaling and metabolism [25], it is very likely that the integration between mechanisms accelerating radical formation and antioxidant systems, able to decrease ROS burden, can represent the strategy to maintain stress levels into appropriate ranges, thus predisposing to a longer and possibly healthy life. In such a scenario, to know the genetic background affecting the degree of oxidative stress and/or the cellular stress response, likewise influencing age-associated traits, can help understand the link between stress response and human aging.

Although several genetic variants affecting directly or not the antioxidant defense mechanisms have been demonstrated to be positively or negatively associated with human longevity (see [25] for a complete gene list), results are often not replicated, and few data are available regarding the effect of stress mediators variability on the quality of aging. When we started the investigation of the association between genetic variants in the oxidative stress pathway and the quality of aging, we selected* TXNRD1* gene, encoding for the endogenous regulator of thioredoxin, as a possible predisposing factor to healthy aging, because of suggestive evidences of a possible role in longevity coming from our previous association studies in the Danish population [14]. Further, in the same Northern European nonagenarian cohort, we demonstrated a correlation of its variability with ADL levels [15], supporting the role of this gene in modulating physical decline, possibly via modulating oxidative stress. However, because nonreplication in different population is one of the most important drawbacks of association studies, we wanted to test the association in a population different from the Danish one, as for genetic background and quality of aging [27].

In the present study, we analyzed the association of 9 tagging SNPs at* TXNRD1* gene with markers of physical and cognitive status, in a Southern-Italian cohort of 588 subjects ranging from 64 to 107 years of age. We found association with geriatric parameters measuring physical/neuromuscular functionality, confirming a major role of this gene on physical status, as well as the absence of correlation with cognitive status, as previously observed [15]. In particular, three SNPs were driving the associations with parameters of physical performance: rs4445711, which was associated with ADL performance and Walking ability, rs11111979 associated with Walking, and rs1128446 associated with Chair stand score. These associations, which remained significant also when correcting for multiple testing (theop < 0.05), were found only in the long lived group, thus being after the 85 years of age in agreement with various studies reporting the age specificity of geriatric biomarkers due to different age-related decline rate of physical functionalities [28]. Although just at a nominal significance level of 0.05, one of the above SNPs, rs1128446, was associated with Chair stand performance also in the control group, thus suggesting an effect of this genetic variability after 64 years of age and onwards.

The evidence of a major role of* TXNRD1* gene after the 85 years of age should suggest an influence of its variability on extreme survival. However, when analyzing the correlation with longevity, we found only a trend of association with rs4964728 and rs7310505.

As for rs7310505, this variant was found associated with ADL levels in Danish nonagenarians [15]. We could not replicate this association in the sample analyzed in this paper, although we observed an association with Walking ability in the younger controls group, not holding multiple comparisons testing. Instead, we confirmed in the Southern sample the negative association with survival previously observed in Northern Europeans; although the results do not hold multiple comparisons in our work, both studies indicate this variant as a possible susceptibility factor for oxidative stress response at old age. A possible explanation of the borderline results of association with survival obtained in this work can be that the sample of nonagenarians here analyzed is one-third of those available in the Northern European cohort (349 older samples versus 1088 Danish nonagenarians). Thus, sample size in Southern Italians should be increased in a future work, to reach statistical significance.

None of the SNPs found associated in the current study have a known functional effect. It is however worthy of note that the* TXNRD1* gene, which displays a complex genomic organization, can undergo extensive alternative splicing, mostly at the 5′ end, giving rise to different protein variants likely having specialized functions in a cell- and tissue-specific manner [29]. Therefore, as most of these SNPs occur in the 5′ intronic region of the gene (Table 1 and Figure S2), they may affect splicing and/or splicing rate. Additional hypothesis is that these SNPs are in LD with variants not yet identified.

In conclusion, results here reported confirm* TXNRD1* as one of the key modulators of stress response associated with physical status at old age, as previously found in Northern Europeans. In particular, the present paper adds a piece of evidence about a consistent effect of* TXNRD1* on health status after 85 years of age in humans. Data here reported cannot be considered exhaustive, especially from a clinical point of view, but they support future studies on the role of the whole thioredoxin pathway in the quality of aging and longevity. In particular an integrated analysis of stress response determinants with those belonging to another crucial pathway in longevity should be applied, for shedding a light on the complex scenario of genetic determinants of human aging and longevity.

## Supplementary Material

There are two figures in supplementary material: in Figure S1, is reported the LD schematic representation (r2 value) in the TXNRD1 gene region, covered by the 9 genotyped SNPs (chr12:103137978-103260828); in Figure S2 is represented the TXNRD1 gene and its different isoforms, with the position of the analyzed SNPs.

## Figures and Tables

**Table 1:** Description of the 10 *TXNRD1* SNPs genotyped on human chromosome 12.

SNP	SNP var.	HapMap position	dbSNP MAF	Genomic location
rs7310815	C/G	103137978	G = 0.284	Intron 1
rs4445711	A/G	103160731	G = 0.287	Intron 1
rs4964728	A/G	103173863	G = 0.194	Intron 2
rs7310505	A/C	103178678	A = 0.248	Intron 3
rs10778318	A/G	103195636	A = 0.293	Intron 3
rs11111979	C/G	103204912	G = 0.476	Intron 3′–5′UTR
rs1128446	C/G	103204972	G = 0.183	Intron 3′–5′UTR
rs7962423	A/G	103235040	G = 0.108	Intron 7
rs17202060	C/T	103254976	T = 0.359	Intron 15
rs10861203	A/G	103260828	A = 0.184	Intron 16

*Note*. MAF: minor allele frequency.

**Table 2:** Sociodemographic characteristics and functional parameters in the sample stratified for group membership.

	Younger controls	Long lived
	(*n* = 239)	(*n* = 349)
Age (year)		
Mean (SD)	73.89 (5.66)	95.26 (4.02)
Range	64–85	86–107
MMSE		
Normal (≥23)	162	39
Impaired (<23)	74	276
HG strength		
Mean (SD)	22.19 (9.11)	13.55 (6.38)
Range	4–55	1–42
ADL^∗^ [*n* (%)]		
Nondisable (=5)	204 (85.4%)	121 (34.7%)
Disable (<5)	35 (14.6%)	228 (65.3%)
Chair stand test		
Mean (SD)	16.60 (5.53)	21.20 (6.75)
Range	2–45	8–43
Walking 500 meters [*n* (%)]		
Yes	187 (78.2%)	120 (35.6)
No	52 (21.8%)	217 (64.4%)
BMI		
Mean (SD)	27.28 (4.28)	23.57 (4.11)
Range	15.01–41.78	12.98–40.54

^∗^Participants were defined as “not disabled” if independent in all items and “disabled” if dependent in at least one item.

MMSE: Mini Mental State Examination; ADL: Activity of Daily Living; HG: Hand Grip.

**Table 3:** *TXNRD1* SNPs showing at least one significant association with functional parameters under a nominal level (*p* ^Model^ < 0.05), in the sample stratified by age class, as obtained by RobustSNP association test.

SNP	Sample	Functional parameters	*z*-score	*p* ^Model^	theop
rs4445711	Long lived	ADL	2.279	0.022 (R)	0.049
	Long lived	Walking	−2.643	0.008 (R)	0.018

rs11111979	Long lived	Walking	−2.341	0.019 (R)	0.042

rs10861203	Long lived	HG	1.985	0.047 (D)	0.099

rs1128446	Long lived	Chair stand	−2.296	0.021 (R)	0.046
	Younger controls	Chair stand	−2.157	0.030 (R)	0.064

rs7310505	Younger controls	Walking	−2.085	0.037 (A)	0.078

*z*-score represents the *z*-statistics for the regression analyses; *p* ^Model^ is the *p* value of the best genetic model, where R is recessive, D is dominant, and A is additive model. theop refers to the *p* value adjusted for multiple comparisons (due to the three different tested genetic models).

**Table 4:** Results of the RobustSNP association test with longevity in the analyzed sample.

SNP	MAF	*z*-score	*p* ^Model^	theop
rs7310815	G = 0.107	0.900	0.367 (D)	0.602
rs4964728	G = 0.203	−2.037	0.041 (A)	0.087
rs7310505	A = 0.260	−1.795	0.072 (A)	0.147
rs10778318	A = 0.333	−0.330	0.741 (D)	0.932
rs11111979	C = 0.454	0.626	0.530 (R)	0.778
rs1128446	G = 0.220	1.079	0.280 (D)	0.485
rs17202060	T = 0.383	1.283	0.199 (R)	0.361
rs10861203	A = 0.252	0.398	0.690 (A)	0.904

Model refers to the most likely genetic model among the dominant, recessive, and additive ones. *p* ^Model^ refers to the *p* value for the most likely genetic model. theop refers to the *p* value adjusted for multiple comparisons (due to the three different tested genetic models).

*z*-score represents the *z*-statistics for the regression analyses; the variable sex was considered a covariate.

MAF: minor allele frequency.
