# Supplementary material for: Antioxidants and Quality of Aging: Further Evidences for a Major Role of TXNRD1 Gene Variability on Physical Performance at Old Age
Source: Oxid Med Cell Longev. 2015 Apr 29;2015:926067. doi: 10.1155/2015/926067 (PMC4429211; doi:10.1155/2015/926067)
Supplement: Supplementary file 1 — There are two figures in supplementary material: in Figure S1, is reported the LD schematic representation (r2 value) in the TXNRD1 gene region, covered by the 9 genotyped SNPs (chr12:103137978-103260828); in Figure S2 is represented the TXNRD1 gene and its different isoforms, with the position of the analyzed SNPs. [file 926067.f1.zip › 926067.f1/926067.r2.attachment.docx]

**Table S1** Complete results of the RobustSNP association test with the functional parameters in the sample stratified by age class.

Significant associations under a nominal level (p<0.05) are indicated in bold.

a) ADL

| SNP | Controls (N=239) | | | | Cases (N=349) | | | |
| --- | --- | --- | --- | --- | --- | --- | --- | --- |
|  | z-score | pModel | Model | theoP | z-score | pModel | Model | theoP |
| rs7310815 | -1,547 | 0,121 | recessive | 0,233 | -1,569 | 0,116 | recessive | 0,225 |
| **rs4445711** | 1,086 | 0,277 | additive | 0,480 | **2,279** | **0,022** | **recessive** | **0,049** |
| rs4964728 | -0,911 | 0,362 | dominant | 0,588 | 0,341 | 0,732 | dominant | 0,929 |
| rs7310505 | -0,940 | 0,346 | dominant | 0,565 | 0,333 | 0,738 | dominant | 0,932 |
| rs10778318 | -0,903 | 0,366 | dominant | 0,591 | 0,901 | 0,367 | recessive | 0,604 |
| rs11111979 | -0,355 | 0,722 | recessive | 0,921 | -0,785 | 0,431 | dominant | 0,677 |
| rs1128446 | -0,505 | 0,613 | recessive | 0,852 | -1,473 | 0,140 | dominant | 0,270 |
| rs17202060 | 0,279 | 0,780 | recessive | 0,949 | 1,450 | 0,146 | additive | 0,276 |
| rs10861203 | -0,988 | 0,323 | additive | 0,548 | -1,644 | 0,100 | dominant | 0,199 |

* Covariate: sex, age

b) HG

| SNP | Controls (N=226) | | | | Cases (N=307) | | | |
| --- | --- | --- | --- | --- | --- | --- | --- | --- |
|  | z-score | pModel | Model | theoP | z-score | pModel | Model | theoP |
| rs7310815 | 1,344 | 0,178 | recessive | 0,329 | -0,752 | 0,451 | dominant | 0,700 |
| rs4445711 | 1,261 | 0,207 | recessive | 0,380 | -0,612 | 0,539 | dominant | 0,783 |
| rs4964728 | -1,756 | 0,078 | recessive | 0,155 | 1,123 | 0,261 | recessive | 0,461 |
| rs7310505 | -1,279 | 0,200 | recessive | 0,360 | -0,838 | 0,401 | additive | 0,634 |
| rs10778318 | 1,840 | 0,065 | dominant | 0,133 | -1,492 | 0,135 | recessive | 0,262 |
| rs11111979 | 1,863 | 0,062 | dominant | 0,128 | 0,730 | 0,464 | dominant | 0,707 |
| rs1128446 | 1,653 | 0,098 | additive | 0,192 | 1,870 | 0,061 | dominant | 0,127 |
| rs17202060 | -1,384 | 0,166 | recessive | 0,302 | -0,722 | 0,470 | recessive | 0,706 |
| **rs10861203** | 0,624 | 0,532 | dominant | 0,777 | **1,985** | **0,047** | **dominant** | **0,099** |

* Covariate: sex, age, height

c) Chair stand

| SNP | Controls (N=201) | | | | Cases (N=173) | | | |
| --- | --- | --- | --- | --- | --- | --- | --- | --- |
|  | z-score | pModel | Model | theoP | z-score | pModel | Model | theoP |
| rs7310815 | -1,422 | 0,154 | recessive | 0,292 | 0,806 | 0,419 | dominant | 0,666 |
| rs4445711 | -1,409 | 0,158 | additive | 0,292 | -0,648 | 0,516 | recessive | 0,770 |
| rs4964728 | -0,666 | 0,505 | additive | 0,756 | -1,331 | 0,183 | recessive | 0,342 |
| rs7310505 | -1,717 | 0,085 | recessive | 0,171 | 1,054 | 0,291 | dominant | 0,500 |
| rs10778318 | -1,763 | 0,077 | additive | 0,156 | -0,675 | 0,499 | additive | 0,748 |
| rs11111979 | -1,185 | 0,235 | additive | 0,421 | 0,702 | 0,482 | additive | 0,727 |
| **rs1128446** | **-2,157** | **0,030** | **recessive** | **0,064** | **-2,296** | **0,021** | **recessive** | **0,046** |
| rs17202060 | 0,904 | 0,365 | recessive | 0,586 | 0,229 | 0,818 | dominant | 0,966 |
| rs10861203 | -1,913 | 0,055 | recessive | 0,114 | -1,513 | 0,130 | recessive | 0,250 |

* Covariate: sex, age

d) Walking

| SNP | Controls (N=239) | | | | Cases (N=332) | | | |
| --- | --- | --- | --- | --- | --- | --- | --- | --- |
|  | z-score | pModel | Model | theoP | z-score | pModel | Model | theoP |
| rs7310815 | 1,716 | 0,086 | recessive | 0,170 | 0,145 | 0,884 | additive | 0,984 |
| **rs4445711** | -0,227 | 0,819 | recessive | 0,967 | **-2,643** | **0,008** | **recessive** | **0,018** |
| rs4964728 | 0,152 | 0,878 | dominant | 0,984 | -0,404 | 0,685 | additive | 0,902 |
| **rs7310505** | **-2,085** | **0,037** | **additive** | **0,078** | -1,466 | 0,142 | dominant | 0,272 |
| rs10778318 | -1,544 | 0,122 | recessive | 0,232 | -1,060 | 0,289 | recessive | 0,499 |
| **rs11111979** | 0,418 | 0,675 | dominant | 0,894 | **-2,341** | **0,019** | **recessive** | **0,042** |
| rs1128446 | -0,301 | 0,763 | dominant | 0,944 | 1,062 | 0,287 | recessive | 0,495 |
| rs17202060 | -1,107 | 0,267 | recessive | 0,460 | -1,419 | 0,155 | dominant | 0,289 |
| rs10861203 | -0,571 | 0,567 | recessive | 0,814 | 1,199 | 0,230 | recessive | 0,413 |

* Covariate: sex, age

e) MMSE

| SNP | Controls (N=236) | | | | Cases (N=315) | | | |
| --- | --- | --- | --- | --- | --- | --- | --- | --- |
|  | z-score | pModel | Model | theoP | z-score | pModel | Model | theoP |
| rs7310815 | 0,453 | 0,650 | dominant | 0,878 | -0,467 | 0,640 | recessive | 0,869 |
| rs4445711 | 0,803 | 0,421 | dominant | 0,669 | -0,975 | 0,329 | recessive | 0,546 |
| rs4964728 | -0,819 | 0,412 | dominant | 0,657 | 1,620 | 0,105 | recessive | 0,203 |
| rs7310505 | -0,533 | 0,593 | dominant | 0,833 | 0,281 | 0,778 | recessive | 0,951 |
| rs10778318 | -1,387 | 0,165 | dominant | 0,307 | 0,366 | 0,714 | recessive | 0,918 |
| rs11111979 | 0,339 | 0,733 | recessive | 0,928 | -0,387 | 0,698 | recessive | 0,907 |
| rs1128446 | -1,926 | 0,054 | recessive | 0,112 | 0,994 | 0,320 | recessive | 0,546 |
| rs17202060 | -0,961 | 0,336 | dominant | 0,559 | -1,431 | 0,152 | additive | 0,284 |
| rs10861203 | -1,692 | 0,090 | additive | 0,181 | -0,710 | 0,477 | dominant | 0,730 |

* Adjusted as reported in Grigoletto et al., 1999

Model refers to the most likely genetic model among the dominant, recessive and additive ones. pmodel refers to the p value for the most likely genetic model. theoP refers to the p value adjusted for multiple comparisons (due to the three different tested genetic models).

z-score represents the z-statistics for the regression analyses; MAF minor allele frequency
