# Supplementary material for: Antioxidants and Quality of Aging: Further Evidences for a Major Role of TXNRD1 Gene Variability on Physical Performance at Old Age
Source: Oxid Med Cell Longev. 2015 Apr 29;2015:926067. doi: 10.1155/2015/926067 (PMC4429211; doi:10.1155/2015/926067)
Supplement: Supplementary file 1 — There are two figures in supplementary material: in Figure S1, is reported the LD schematic representation (r2 value) in the TXNRD1 gene region, covered by the 9 genotyped SNPs (chr12:103137978-103260828); in Figure S2 is represented the TXNRD1 gene and its different isoforms, with the position of the analyzed SNPs. [file 926067.f1.zip › 926067.f1/mat.926067.v2.pdf]

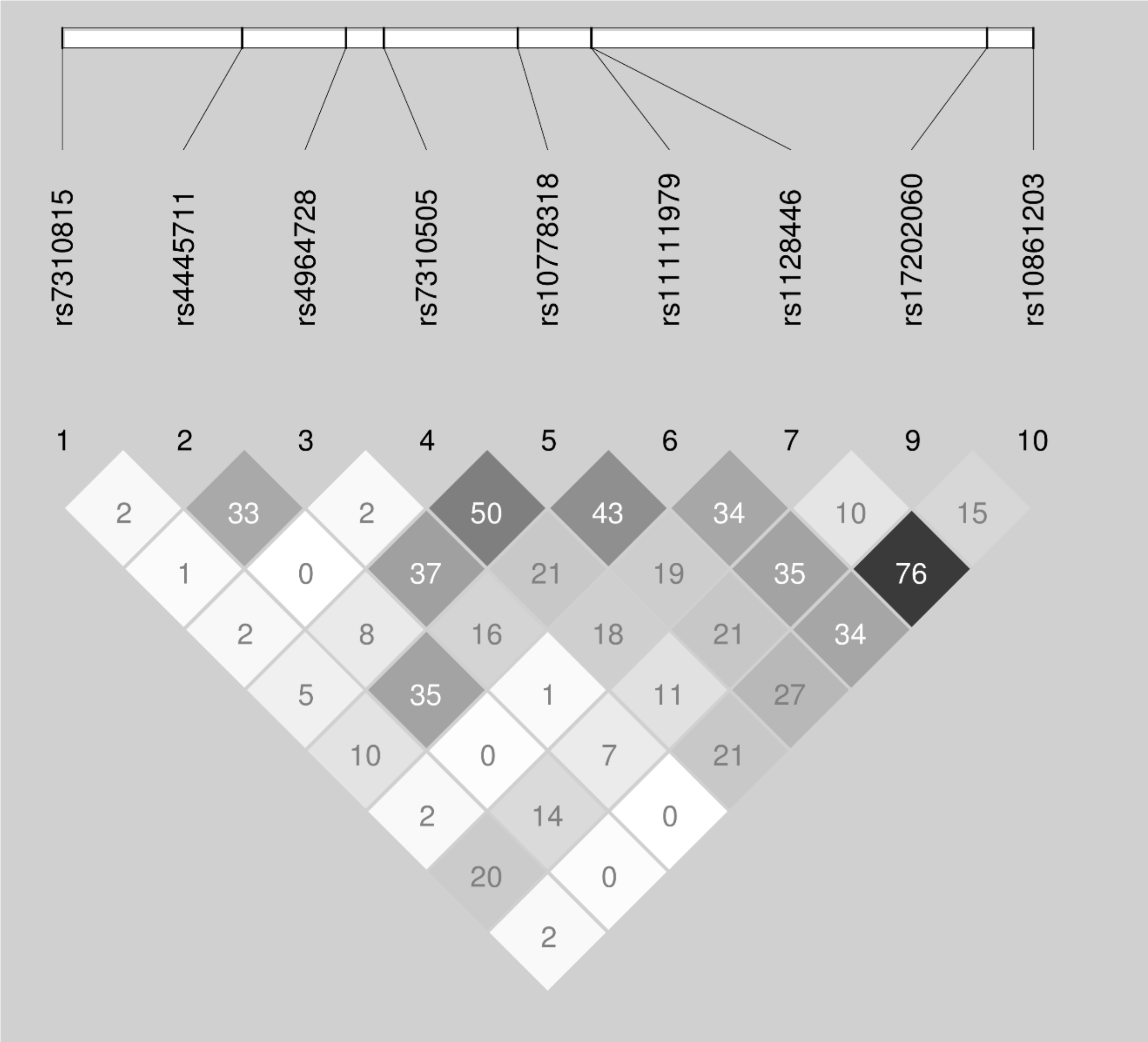

Figure S1. LD schematic representation (r2 value) in the TXNRD1 gene region covered by the 9 SNPs genotyped (chr12: 103137978- 103260828).

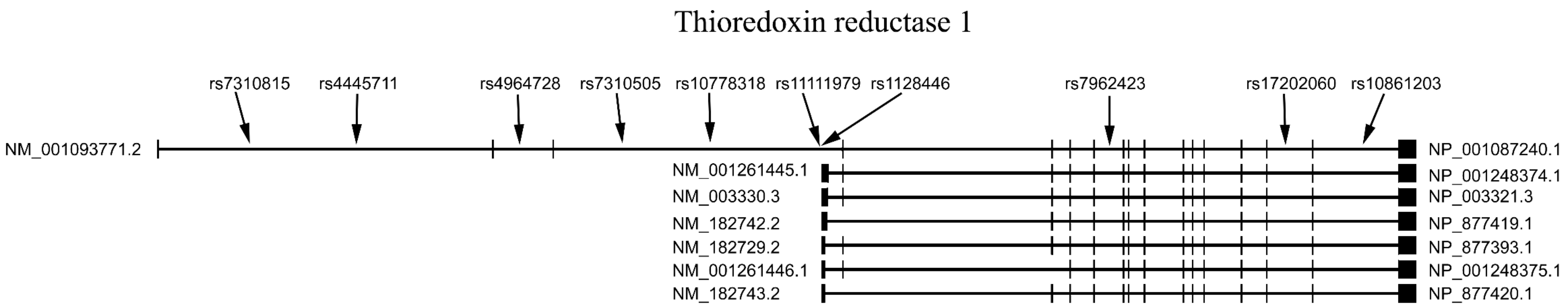

Figure S2. Representation of thioredoxin reductase 1 gene and different isoforms. The position of different analyzed SNPs are reported.
